# Manganese-based Materials Inspired by Photosynthesis for Water-Splitting

**DOI:** 10.3390/ma4101693

**Published:** 2011-09-28

**Authors:** Harvey J.M. Hou

**Affiliations:** Department of Physical Sciences, Alabama State University, Montgomery, AL 36104, USA; E-Mail: hhou@alasu.edu; Tel.: +1-334-229-5121; Fax: +1-334-229-5409.

**Keywords:** photosynthesis, manganese, photosystem II, artificial photosynthesis, semiconductor, Nafion

## Abstract

In nature, the water-splitting reaction via photosynthesis driven by sunlight in plants, algae, and cyanobacteria stores the vast solar energy and provides vital oxygen to life on earth. The recent advances in elucidating the structures and functions of natural photosynthesis has provided firm framework and solid foundation in applying the knowledge to transform the carbon-based energy to renewable solar energy into our energy systems. In this review, inspired by photosynthesis robust photo water-splitting systems using manganese-containing materials including Mn-terpy dimer/titanium oxide, Mn-oxo tetramer/Nafion, and Mn-terpy oligomer/tungsten oxide, in solar fuel production are summarized and evaluated. Potential problems and future endeavors are also discussed.

## 1. Introduction

In nature, oxygenic photosynthesis harvests solar energy from the sun using a range of photosynthetic antenna systems and funnels the excitation energy to the reaction centers, where the light energy is stored and converted into chemical energy and oxygen as a byproduct using by water splitting reaction in plants, algae, and cyanobacteria [1,2]. Recent progresses to understand the natural photosynthesis at the molecular level have been acquired [3,4] and provides a solid basis for unraveling the mechanisms of water splitting and O-O bond formation, which is one of nature’s most fascinating and important chemical reactions. The knowledge inspires and facilitates the creation of artificial photosynthetic model systems [5,6,7,8,9,10]. A significant amount of research efforts has been placed toward the development of artificial catalytic systems composed of molecular and supramolecular architectures and discussed in several relevant reviews [4,11,12,13,14,15,16,17]. In particular, the synthesis and characterization of Mn-containing compounds such as CaMn_3_O_4_, CaMn_2_O_4_, multiple phases of MnO_2_, and Mn_3_O_4_ are fascinating [18,19,20]. These synthetic models will provide a deeper understanding of the PS II mechanism and complex cluster assembly and open a new window in the design of better molecular catalytic systems for water splitting. Due to the space limitation, the synthesis and characterization of the Mn-containing compounds to mimic the structure of PS II is beyond the scope of this review and will not be discussed. 

In this review, manganese-based catalytic systems including Mn-terpy dimer/titanium oxide, Mn-oxo tetramer/Nafion, Mn-terpy oligomer/tungsten oxide, which are highly active in water oxidation catalysis, will be summarized and evaluated in terms of renewable energy production. Potential problems and future endeavors are also presented. 

## 2. Photosynthetic Water Splitting

Photosystem II (PS II) is the site of photosynthetic water oxidation and a large protein complex containing more than 20 subunits with a molecular mass of about 350 kDa. Much of its protein and pigment components control the light harvesting and transferring of photonic energy, and the energy conversion via oxidation of water is carried out at a cluster of metals, Mn_4_CaO_5_ center, in the oxygen-evolving complex (OEC). When four electron and four protons are extracted from two molecules of water, one molecule of dioxygen is formed. The mechanism of photosynthetic water oxidation in PS II is believed to occur through four distinct oxidation steps as the S-state cycle by Kok *et al.* and Joliot *et al.* [21,22]. 

**Figure 1:**
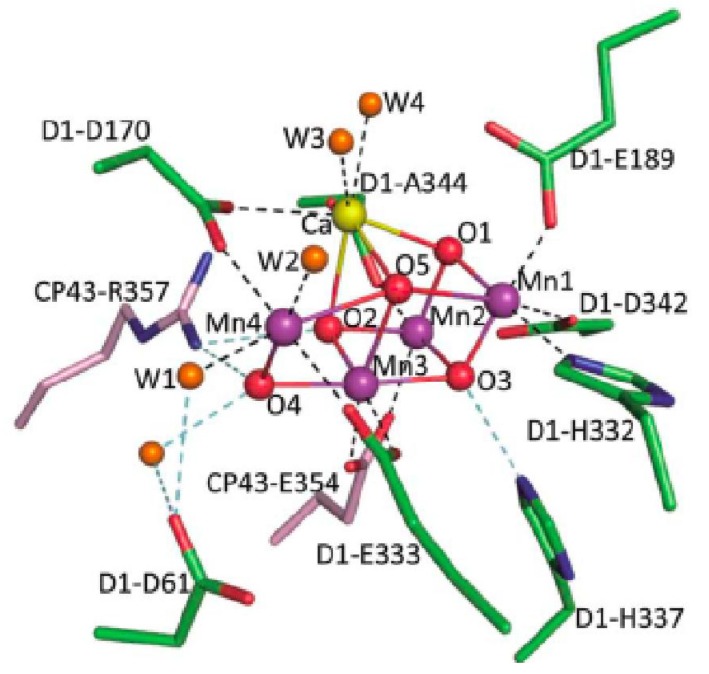
Structure of the Mn_4_CaO_5_ cluster and its ligand environment in photosystem II oxygen-evolving complex at a resolution of 1.9 A. (from reference [28], produced with permission from Macmillan Publisher).

The structure of PS II has been solved at resolutions of 2.9–3.8 A from thermophilic cyanobacteria, *Thermosynechococcus elongates* and *Thermosynechococcus vulcanus* [23,24,25,26,27]. However, the resolution is not high enough to reveal the structure of Mn cluster and subtract water molecules. A recent report determines the high-resolution structure of PS II at a resolution of 1.9 A from *Thermosynechococcus vulcanus* [28]. The structure reveals the complete geometry arrangement of the Mn_4_CaO_5_ cluster including its oxo bridges and ligands (Figure 1). Of these five metals and five oxygen atoms, three manganese (Mn1 to Mn3), one calcium and four oxygen atoms for a cubane-like structure. Owing to the difference in bond lengths, the Mn_3_CaO_4_ cubane is not symmetric. The fourth manganese (Mn4) is located outside the cubane and is linked to Mn1 and Mn3. Every two adjacent manganese atoms are linked by di-μ-oxo bridge. The calcium is linked to all four manganese by oxo-bonds, either by d-μ-oxo or mono-μ-oxo bridges. 

Four water molecules (W1 to W4) are also identified to be associated with the Mn_4_CaO_5_ cluster. W1 and W2 are coordinated to Mn4, and W3 and W4 are linked to calcium. No water molecules were found to associate with Mn1, Mn2, and Mn3. All of the amino-acid residues coordinated to the Mn_4_CaO_5_ cluster were identified and divided into two layers: (1) direct ligands to the cluster include D1-Glu 189, D1-Asp 170, D1-Glu 333, D1-Asp 342, CP43-Gllu 354, D1-His 332, D1-Ala 334; and (2) second coordination sphere including D1-Asp 61, D1-His 337 and CP43-Arg 357. Each of manganese has six ligands whereas the calcium has seven ligands. These pattern and geometric positions of the metal atoms may have important function for the mechanisms of water splitting and O-O bond formation. The important finding of the new Mn_4_CaO_5_ structure is apparent longer distances between O5 and metal atoms suggest the corresponding bonds are weak. Therefore, O5 may have a lower negative charge that the normal oxygen atoms and is likely a hydroxide ion in the S1 state. As the W2 and W3 are within the hydrogen-bond distances to O5 and may be the substrate of water oxidation reaction.

## 3. Mn-based Catalyst and Their Mechanisms

### 3.1. Mechanism of Photosynthetic Water Splitting 

The four-oxidation steps with five intermediates known as S-states are required for water oxidation; however, there is limited information at the molecular level. Brudvig *et al.* proposed a possible “molecular” model based on a structural conversion of a Mn_4_O_6_ “cubane”-like complex [29]. In this model, a tetrameric manganese site in PS II is sequentially oxidized. Each manganese ion in the tetrameric complex is proposed to be coordinated to the protein via N or O ligand to form an octahedral coordination. For the lower oxidation states (S_0_-S_2_), the manganese complex is in a cubane structure. Upon reaching the S_3_ state, the complex may for a Mn_4_O_6_ adamantane structure. The Dismukes and Christou groups proposed a “butterfly or double pivot mechanism [30,31]. In this model, oxygen is evolved by O-O bond formation across a face of the cube and subsequent loss of O_2_ free two of the manganese atoms and open the cube. This idea was supported by gas phase experiments [31]. 

**Figure 2:**
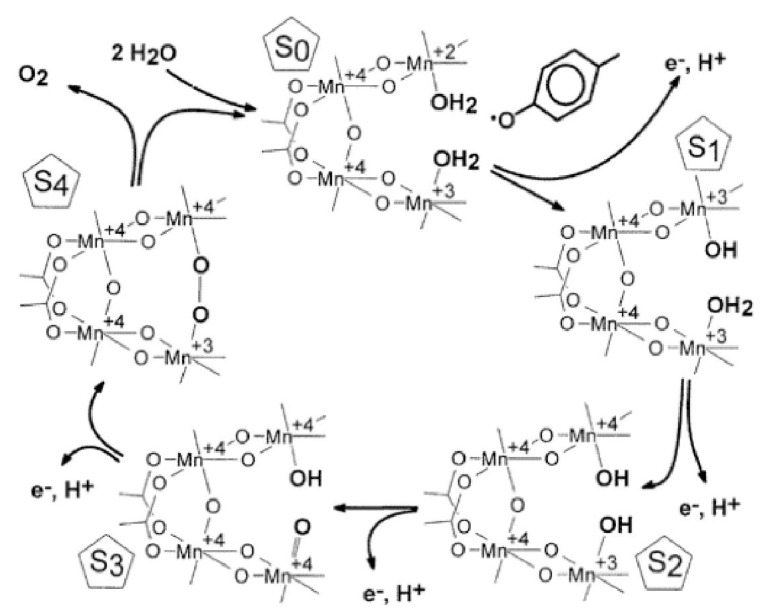
Proposed photosynthetic oxygen evolution mechanistic model (from [62], produced with permission from the American Association for the Advancement of Science).

Figure 2 showed a popular mechanism known as “2 + 2” model proposed by Babcock *et al.* This model assumes that the structure of Mn center of PS II is a dimer-of-dimer. Each of the two dimers has water ligated in close proximity. The water molecules are progressively oxidized coupled with proton release until a manganese-oxo group formation on one of dimer and production of hydroxide ion on the other in three steps (S_0_→S_1_→S_2_→S_3_ transitions). The O-O bond formation with a quick release of O_2_ (S_4_→S_0_) is followed by the removal of the last proton and generation of a peroxo species between the two dimers (S_3_→S_4_). Note: the new structure of PS II does not support the structure of “dimer of dimer”. Although the concept of the proposal is valid, the detailed mechanism of PS II water oxidation is unknown and still under debate. 

The key point for the proposals of water oxidation cycle in photosynthesis is the formation of a high-valent metal oxo group, which is produced by the nucleophilic attack by a near water or hydroxide group. Another area of debate is the nature of high-valent manganese-oxo species. Current evidence cannot rule out the possibility of mechanisms involving manganese-oxo or manganese-oxyl species, we refer to this species as a Mn(V)-oxo intermediate.

### 3.2. Mn-oxo tetramer/Nafion and Mn-oxo tetramer/TiO_2_ System

PS II is a very challenging protein to study by biophysical methods. One of the reasons is that PS II OEC is extremely sensitive and vulnerable to environment and experimental conditions. It is well documented that PS II OEC is the target of UV and blue region light radiation [32,33,34,35,36]. The photodamage of PS II is triggered via the direct absorption of a photon by manganese ions in the Mn_4_Ca center, as evidenced by the action spectra of PS II photoinhibition and synthetic manganese-oxo dimer compound [34,35]. The UV photoinhibition at the Mn cluster is likely involved the formation of a high-valent manganese species [34,37]. In addition the Mn_4_Ca active site in the single crystal of PS II OEC can be damaged by exposure to X-ray radiation revealed by X-ray absorption spectroscopy. [38]

To elucidate the details of the OEC reaction mechanism, bioinorganic chemists have made new complexes to mimic the proposed biological mechanisms. The study of model complexes greatly improved our understanding of the natural water-oxidation mechanism. The design of functional oxygen evolving compounds is successfully achieved. One such example is Mn-oxo tetramer cubic structure developed by Dismukes and co-workers [31]. The key feature of the compound, [Mn_4_O_4_(dpp)_6_], is its cubical [Mn_4_O_4_]^n+^ core, surrounded by six bridging bidentate chelates to the manganese ions. The water oxidation reaction proceeds with high quantum efficiency (>50%) in the gas phase, and the O_2_ product is exclusively produced from the corner oxo’s of the cube. The cubane core facilitates both the selective reduction of two of the four oxygen bridges to water molecules and their photorearrangement to release an O_2_ molecule upon photoexcitation at 350 nm. The highest oxidation state observed in the cubane family contains the [Mn_4_O_4_]^7+^ core, or [Mn_4_O_4_L_6_]^+^.

**Figure 3:**
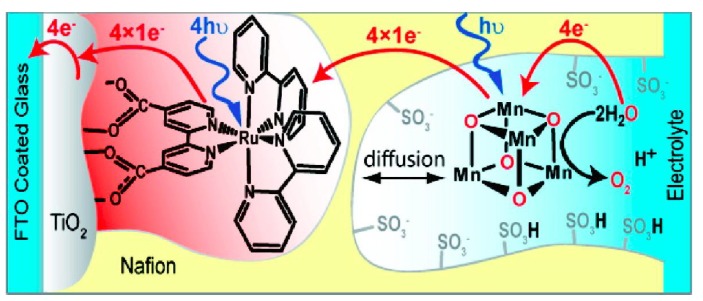
Mn-tetramer/Nafion water splitting catalytic system. Photoanode are made of a titania layer sensitized with the dye, [Ru(bipy)_2_(bipy(COO)_2_], and a Nafion film doped with the Mn-oxo tetramer, [Mn_4_O_4_L_6_]^+^. (From reference [9], produced with permission from American Chemical Society).

Due to the insolubitity of cubium in water and most organic solvents, Mn-oxo tetramer/Nafion system was developed by depositing the Mn-oxo tetramer into a thin Nafion membrane on a conducting electrode. As shown in Figure 3, the system is a robust catalyst that sustains the photoelectrooxidation of water to O_2_ [39]. The perfluorinated polymer Nafion comprises hydrophobic domains and provides excellent aqueous channels for proton transfer in water oxidation reaction. The photooxidation of water is likely due to a catalytically active species originating from the photolysis of Mn-oxo tetramer which is bonded to or activated by the sulfonate groups of the aqueous Nafion membrane. The authors postulate that the Mn-oxo tetramer/Nafion follows the similar photoreaction mechanism of Mn-tetramer in gas phase with O_2_ being released upon photodissociation to yield the “reduced butterfly species” [Mn_4_O_2_L_5_]^2+^. This species may be more stable in restricted hydrophobic pockets of Nafion. 

Recent *in situ* X-ray absorption spectroscopy and transmission electron microscopy revealed that the synthetic Mn-oxo tetramer cluster undergoes a dissociation to form Mn(II) compound in the Nafion matrix [40]. The Mn(II) species can be reoxidized to generate the catalytic Mn(III/V) oxide and close the water oxidation catalytic cycle. The original Mn-oxo tetramer is likely serves only as a precursor to the catalytically active material.

### 3.3. Mn-terpy dimer and Mn-terpy dimer/TiO_2_ System

**Figure 4:**
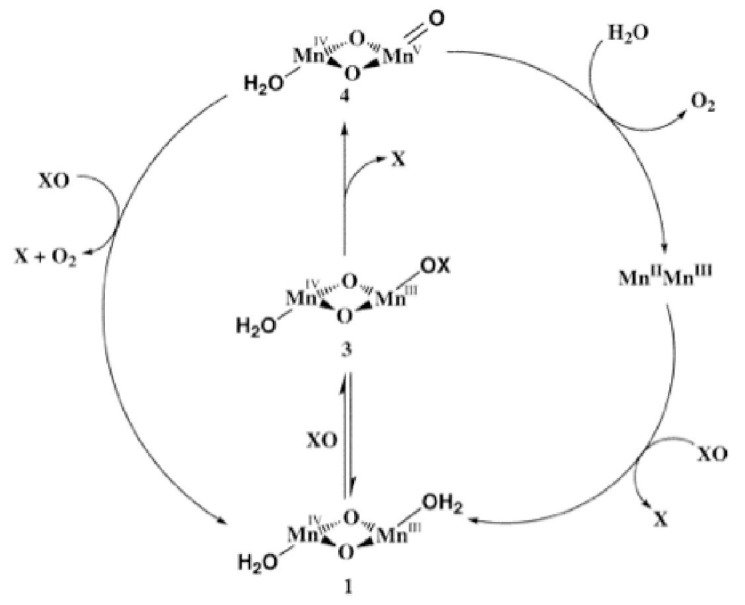
Oxygen evolution mechanism of Mn-terpy dimer in the presence of chemical oxidant, XO. Left pathway: attach of XO on the terminal oxo ligand. Right pathway: attack of solvent water on the terminal oxo ligand. (from reference [42], produced with permission from the American Chemical Society).

Another excellent example is the first PS II functional model for oxygen evolving complex so called “Mn-terpy dimer,” ([OH_2_(terpy)Mn(O)_2_Mn(terpy)OH_2_](NO_3_)_3_•6H_2_O) [41]. Figure 4 showed a proposed mechanism for the formation of the reactive intermediate in oxygen evolution catalytically by the Mn-terpy dimer in the present of chemical oxidants, XO, such as hypochlorite or peroxymonsulfate (Oxone). The XO binds initially to the Mn-terpy dimer reversibly (step 1→3). Once formed, compound 3 can react with XO to form the Mn(V)=O intermediate species (step 3 → 4). This is the rate-limiting step in oxygen evolution. The O-O bond-forming step involves attack of solvent or Oxone on the oxo ligand [42]. 

By mimicking the photoinduced charge separation of natural photosynthesis, molecular and supramolecular model systems can provide important inspiration for practical solar fuel production. Hammarstrom, Styring, and co-workers prepared molecular assemblies coupling Mn complexes with the dye photosensitizer, [Ru(bpy)_3_]^2+^, and observed light-induced manganese oxidation and long-lived charge separation [43]. Brudvig *et al.* have directly deposited the Mn-terpy dimer on the TiO_2_ nanoparticles (NP) and found that Mn-terpy dimer binds to near amorphous TiO_2_ NP and dimerized to form Mn(IV) tetramer. The Mn(IV) tetramer attached to TiO_2_ oxidizes water to oxygen in the presence of Ce^4+^ as the primary one-electron oxidant [44,45,46]. They also [47,48,49]synthesize a derivative of Mn-terpy dimer on the surface of TiO_2_ NPs *in situ* by first attached the precursor Mn(II) monomers through an anchoring ligand on the TiO_2_ NPs and then assembling the Mn-terpy dimer [50]. The resulting Mn-terpy dimer/TiO_2_ is able to reversibly change mixed valent Mn(III/IV) to Mn(IV/IV) state by photoexcitation and interfacial electron injection into the conduction band of TiO_2_ .

### 3.4. Mn-oligomer/WO_3_ System

The weakness of Mn-terpy dimer is that the compound is unstable under elevated temperature [51]. The decomposition of the Mn-terpy dimer in aqueous solution over 60 °C occurred involving a change in Mn valence. Interestingly, the thermal decomposition of Mn-terpy dimer was found to generate a Mn-containing precipitate that retained catalytic oxygen-evolving activity [51]. The solid Mn-containing material binds terpy ligand, judged by elemental analysis and atomic absorption analysis, and is not simple manganese dioxide. The novel Mn-containing precipitate, assigned as a Mn-terpy oligomer, is thermal stable and may be a unique option for constructing catalytic systems in solar fuel production.

When heated over 60 °C, the Mn-terpy oligomer undergoes thermal decomposition and yields a controlled thin layer on a semiconductor such tungsten oxide. The resulting Mn-oligomer/WO_3_ material is able to efficiently split water to generate oxygen using GC and GC/MS under light illumination [6]. The WO_3_ material alone is unstable over prolong period time of water splitting. At pH 7.0, WO_3_ without Mn-terpy oligomer decayed about 60% within 1 h, whereas approximately 4% performance decrease was observed when the Mn-terpy was present for up to 2 h. In addition to O_2_, the amount of H_2_ generated by the photoelectrochemical setup was also measured. The 2:1 ratio of H_2_ and O_2_ generation rate confirms the complete decomposition of H_2_O by the photogenerated charges unambiguously. Another piece of evidence is the ^18^O isotope experiments. H_2_^18^O was used in water splitting experiment. A significant amount of ^16,18^O_2_ in the headspace gas sample was detected in addition to a small amount of ^18,18^O_2_.

**Figure 5:**
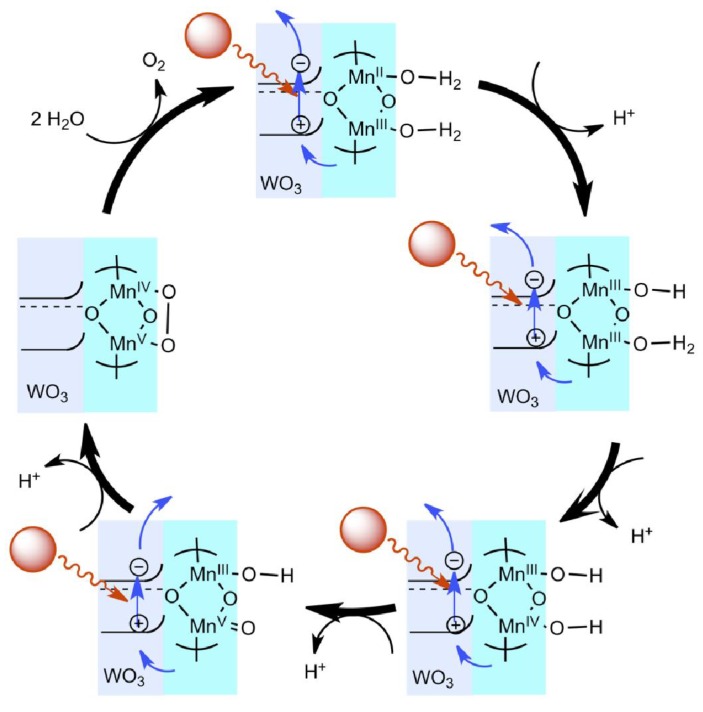
Proposed mechanisms of Mn-terpy oligomer/WO_3_ catalytic system in water oxidation. Black arrows indicate the proton transfer reaction; blue arrows represent the electron transfer steps. Mn-terpy oligomer oxidizes H_2_O oxidation by receiving photogenerated holes from WO_3_ and undergoing a catalytic cycle, in which Mn valance change is a proton-coupled electron transfer in a similar way to PS II oxygen-evolving complex.

Based on the observed experimental data, a possible mechanism of Mn-terpy oligomer/WO_3_ system in water oxidation is proposed in Figure 5. Four light photons are required to oxidize the Mn-terpy species accompanying four proton-coupled steps. At each step, the photon causes charge separation in WO_3_. The hole generated in WO_3_ receives electron from Mn-terpy complex and oxidizes the Mn ion via Mn valence changes. The Mn(V) intermediate species is generated by the fourth photon-driven electron transfer reaction and splits water to dioxygen and regenerate active Mn-terpy catalyst.

## 4. Concluding Remarks

In the recent five years, substantial progress in natural photosynthesis, especially protein structural and mechanistic aspects of PS II oxygen-evolving complex, has inspired the revolutionary development in photoelectrochemical and electrochemical water splitting using manganese-based molecular catalysts as well as Fe-, Co-, Ni-, Ru-, and Ir-containing compounds associated with dye-sensitized semiconductors in solar fuel production [52,53,54,55,56,57,58]. Another highly desirable use for solar energy is powering fuel generation by hydrogen production via water splitting using visible light [59,60]. These catalytic systems mimic the natural photosynthesis and take the advantages of multiple materials with unique properties in terms of light harvesting, energy transfer, charge separation, electron transfer, water oxidation, and hydrogen production. It is highly likely that the progress in the field of nanomaterial and photosynthesis will offer novel technology for transforming the solar fuels into our future sustainable energy sources in the next 10–20 years, hopefully as eventual personalized energy systems [61]. Grand challenges remain, including the discovery of inexpensive, robust, and efficient water-oxidation catalysts. It is worth noting that nanoscale materials are especially appealing in energy research because their dimensions coincide with the length scales of processes fundamental to energy conversion and storage. Future endeavors will be placed on improvement in efficiency and durability using visible light as well as the development of novel catalytic materials involving dye-sensitized solar cells and photoelectrochemical cells.

## References

[1] Barber J. (2009). Photosynthetic energy conversion: Natural and artificial. Chem. Soc. Rev..

[2] Blankenship Robert E., Tiede David M., Barber J., Brudvig Gary W., Fleming G., Ghirardi M., Gunner M.R., Junge W., Kramer David M., Melis A. (2011). Comparing photosynthetic and photovoltaic efficiencies and recognizing the potential for improvement. Science.

[3] Brudvig G.W. (2008). Water oxidation chemistry of photosystem II. Philos. Trans. R. Soc. B.

[4] Barber J., Murray J.W. (2008). Revealing the structure of the Mn-cluster of Photosystem II by X-ray crystallography. Coord. Chem. Rev..

[5] Dismukes G.C., Brimblecombe R., Felton G.A.N., Pryadun R.S., Sheats J.E., Spiccia L., Swiegers G.F. (2009). Development of bioinspired Mn_4_O_4_-cubane water oxidation catalysts: Lessons from photosynthesis. Acc. Chem. Res..

[6] Liu R., Lin Y., Chou L.-Y., Sheehan S.W., He W., Zhang F., Hou H.J.M., Wang D. (2011). Water splitting by tungsten oxide prepared by atomic layer deposition and decoraed with an oxygen-evolving catalyst. Angew. Chem. Int. Ed..

[7] Blakemore J.D., Schley N.D., Balcells D., Hull J.F., Olack G.W., Incarvito C.D., Eisenstein O., Brudvig G.W., Crabtree R.H. (2010). Half-sandwich iridium complexes for homogeneous water-oxidation catalysis. J. Am. Chem. Soc..

[8] Kanan M., Nocera D.G. (2008). *In situ* formation of an oxygen-evolving catalyst in neutral water containing phosphate and Co^2+^. Science.

[9] Brimblecombe R., Koo A., Dismukes G.C., Swiegers G.F., Spiccia L. (2010). Solar-driven water oxidation by a bio-inspired manganese molecular catalyst. J. Am. Chem. Soc..

[10] Zhong D.K., Gamelin D.R. (2010). Photoelectrochemical water oxidation by cobalt catalyst (“Co-Pi”)/α-Fe_2_O_3_ composite photoanodes: Oxygen evolution and resolution of a kinetic bottleneck. J. Am. Chem. Soc..

[11] Dau H., Zaharieva I. (2009). Principles, efficiency, and blueprint character of solar energy conversion in photosynthetic water oxidation. Acc. Chem. Res..

[12] McConnell I., Li G., Brudvig G.W. (2010). Energy conversion in natural and artificial photosynthesis. Chem. Biol..

[13] Cady C.W., Crabtree R.H., Brudvig G.W. (2008). Functional models for the oxygen-evolving complex of photosystem II. Coord. Chem. Rev..

[14] Kanan M.W., Surendranath Y., Nocera D.G. (2009). Cobalt-phosphate oxygen-evolving compound. Chem. Soc. Rev..

[15] Hambourger M., Moore G.F., Kramer D.M., Gust D., Moore A.L., Moore T.A. (2009). Biology and technology for photochemical fuel production. Chem. Soc. Rev..

[16] Kudo A., Miseki Y. (2008). Heterogeneous photocatalyst materials for water splitting. Chem. Soc. Rev..

[17] Lin Y., Yuan G., Liu R., Zhou S., Sheehan S.W., Wang D. (2011). Semiconductor nanostructure-based photoelectrochemical water splitting: A brief review. Chem. Phys. Lett..

[18] Mishra A., Wernsdorfer W., Abboud K.A., Christou G. (2005). The first high oxidation state manganese-calcium cluster: Relevance to the water oxidizing complex of photosynthesis. Chem. Commun..

[19] Kanady J.S., Tsui E.Y., Day M.W., Agapie T. (2011). A synthetic model of the Mn_3_Ca subsite of the oxygen-evolving complex in photosystem II. Science.

[20] Mullins C.S., Pecoraro V.L. (2008). Reflections on small molecule manganese models that seek to mimic photosynthetic water oxidation chemistry. Coord. Chem. Rev..

[21] Joliot P., Barbieri G., Chabaud R. (1969). Model of the system II photochemical centers. Photochem. Photobiol..

[22] Kok B., Forbush B., McGloin M. (1970). Cooperation of charges in photosynthetic O_2_ evolution-I. A linear four step mechanism. Photochem. Photobiol..

[23] Loll B., Kern J., Saenger W., Zouni A., Biesiadka J. (2005). Towards complete cofactor arrangement in the 3.0 A resolution structure of photosystem II. Nature.

[24] Kamiya N., Shen J.R. (2003). Crystal structure of oxygen-evolving photosystem II from *Thermosynechococcus vulcanus* at 3.7-A resolution. Proc. Natl. Acad. Sci. USA.

[25] Ferreira K.N., Iverson T.M., Maghlaoui K., Barber J., Iwata S. (2004). Architecture of the photosynthetic oxygen-evolving center. Science.

[26] Zouni A., Witt H.T., Kern J., Fromme P., Krauss N., Saenger W., Orth P. (2001). Crystal structure of photosystem II from *Synechococcus elongatus* at 3.8 A resolution. Nature.

[27] Guskov A., Kern J., Gabdulkhakov A., Broser M., Zouni A., Saenger W. (2009). Cyanobacterial photosystem II at 2.9 A resolution and the role of quinones, lipid, channels and chloride. Nat. Struct. Mol. Biol..

[28] Umena Y., Kawakami K., Shen J.R., Kamiya N. (2011). Crystal structure of oxygen-evolving photosystem II at a resolution of 1.9 A. Nature.

[29] Brudvig G.W., Crabtree R.H. (1986). Mechanism for photosynthetic O_2_ evolution. Proc. Natl. Acad. Sci. USA.

[30] Christou G. (1989). Manganese carboxylate chemistry and its biological relevance. Acc. Chem. Res..

[31] Ruettinger W., Yagi M., Wolf K., Bernasek S., Dismukes G.C. (2000). O_2_ evolution from the manganese-oxo cubane core Mn_4_O_4_^6+^: A molecular mimic of the photosynthetic water oxidation enzyme?. J. Am. Chem. Soc..

[32] Renger G., Volker M., Eckert H.J., Fromme R., Hohm-veit S., Graber P. (1989). On the mechanism of photosystem II deterioration by UV-B irradiation. Photochem. Photobiol..

[33] Ohnishi N., Allakhverdiev S.I., Takahashi S., Higashi S., Watanabe M., Nishiyama Y., Murata N. (2005). Two-step mechanism of photodamage to photosystem II: Step 1 occurs at the oxygen-evolving complex and step 2 occurs at the photochemical reaction center. Biochemistry.

[34] Wei Z., Cady C., Brudvig G.W., Hou H.J.M. (2011). Photodamage of a Mn(III/IV)-oxo mix valence compound and photosystem II complexes: Evidence that high-valent manganese species is responsible for UV-induced photodamage of oxygen evolving complex in photosystem II. J. Photochem. Photobiol. B.

[35] Hakala M., Tuominen I., Keranen M., Tyystjarvi T., Tyystjarvi E. (2005). Evidence for the role of the oxygen-evolving manganese complex in photoinhibition of Photosystem II. Biochim. Biophys. Acta.

[36] Vass I., Sass L., Spetea C., Bakou A., Ghanotakis D.F., Petrouleas V. (1996). UV-B-induced inhibition of photosystem II electron transport studied by EPR and chlorophyll fluorescence. Impairment of donor and acceptor side components. Biochemistry.

[37] Antal A., Lo W., Armstrong William H. (2009). Illumination with ultraviolet or visible light induces chemical changes in the water soluble manganese complex, [Mn_4_O_6_(bpea)_4_]Br_4_. Photochem. Photobiol..

[38] Yano J., Kern J., Irrgang K.-D., Latimer M.J., Bergmann U., Glatzel P., Pushkar Y., Biesiadka J., Loll B., Sauer K. (2005). X-ray damage to the Mn_4_Ca complex in single crystals of photosystem II: A case study for metalloprotein crystallography. Proc. Natl. Acad. Sci. USA.

[39] Brimblecombe R., Swiegers G.F., Dismukes G.C., Spiccia L. (2008). Sustained water oxidation photocatalysis by a bioinspired manganese cluster. Angew. Chem., Int. Ed..

[40] Hocking R.K., Brimblecombe R., Chang L.-Y., Singh A., Cheah M.H., Clover C., Casey W.H., Spiccia L. (2011). Water-oxidation catalysis by manganese in a geochemical-like cycle. Nat. Chem..

[41] Limburg J., Vrettos J.S., Liable-Sands L.M., Rheingold A.L., Crabtree R.H., Brudvig G.W. (1999). A functional model for O-O bond formation by the O_2_-evolving complex in photosystem II. Science.

[42] Limburg J., Vrettos J.S., Chen H., de Paula J.C., Crabtree R.H., Brudvig G.W. (2001). Characterization of the O_2_-evolving reaction catalyzed by [(terpy)(H_2_O)Mn(III)(O)_2_Mn(IV)(OH_2_)(terpy)](NO_3_)_3_ (terpy = 2,2':6,2"-terpyridine). J. Am. Chem. Soc..

[43] Hammarstrom L., Styring S. (2008). Coupled electron transfers in artificial photosynthesis. Phil. Trans. R. Soc. B.

[44] McNamara W.R., Milot R.L., Song H.-E., Snoeberger R.C., Batista V.S., Schmuttenmaer C.A., Brudvig G.W., Crabtree R.H. (2010). Water-stable, hydroxamate anchors for functionalization of TiO_2_ surfaces with ultrafast interfacial electron transfer. Energy Environ. Sci..

[45] Li G., Sproviero Eduardo M., Snoeberger R., Iguchi N., Black J.D., Crabtree George W., Brudvig Gary W., Batisa V.S. (2009). Deposition of an oxomanganese water oxidation catalyst on TiO_2_ nanoparticles: Computational modeling, assembly and characterization. Energy Environ. Sci..

[46] McNamara W.R., Snoeberger R.C., Li G., Schleicher J.M., Cady C.W., Poyatos M., Schmuttenmaer C.A., Crabtree R.H., Brudvig G.W., Batista V.S. (2008). Acetylacetonate anchors for robust functionalization of TiO_2_ nanoparticles with Mn(II)-terpyridine complexes. J. Am. Chem. Soc..

[47] Youngblood W.J., Lee S.-H.A., Kobayashi Y., Hernandez-Pagan E.A., Hoertz P.G., Moore T.A., Moore A.L., Gust D., Mallouk T.E. (2009). Photoassisted overall water splitting in a visible light-absorbing dye-sensitized photoelectrochemical cell. J. Am. Chem. Soc..

[48] Woodhouse M., Parkinson B.A. (2008). Combinatorial approaches for the identification and optimization of oxide semiconductors for efficient solar photoelectrolysis. Chem. Soc. Rev..

[49] Zhong D.K., Sun J., Inumaru H., Gamelin D.R. (2009). Solar water oxidation by composite catalyst/α-Fe_2_O_3_ photoanodes. J. Am. Chem. Soc..

[50] Li G., Sproviero E.M., McNamara W.R., Snoeberger R.C., Crabtree R.H., Brudvig G.W., Batista V.S. (2010). Reversible visible-light photooxidation of an oxomanganese water-oxidation catalyst covalently anchored to TiO_2_ nanoparticles. J. Phys. Chem. B.

[51] Zhang F., Cady C.W., Brudvig Gary W., Hou H.J.M. (2011). Thermal stability of [Mn(III)(O)_2_Mn(IV)(H_2_O)_2_(Terpy)_2_](NO_3_)_3_ (Terpy = 2,2':6',2"-terpyridine) in aqueous solution. Inorg. Chim. Acta.

[52] Lin Y., Yuan G., Liu R., Zhou S., Sheehan S.W., Wang D. (2011). Nanonet-based hematite for efficient solar water splitting. J. Am. Chem. Soc..

[53] Dinca M., Surendranath Y., Nocera Daniel G. (2010). Nickel-borate oxygen-evolving catalyst that functions under benign conditions. Proc. Natl. Acad. Sci. USA.

[54] Kanan M.W., Yano J., Surendranath Y., Dinca M., Yachandra V.K., Nocera D.G. (2010). Structure and valency of a cobalt-phosphate water oxidation catalyst determined by *in situ* X-ray spectroscopy. J. Am. Chem. Soc..

[55] McAlpin J.G., Surendranath Y., Dinca M., Stich T.A., Stoian S.A., Casey W.H., Nocera D.G., Britt R.D. (2010). EPR evidence for Co(IV) species produced during water oxidation at neutral pH. J. Am. Chem. Soc..

[56] Concepcion J.J., Jurss J.W., Brennaman M.K., Hoertz P.G., Patrocinio A.O.T., Murakami Iha N.Y., Templeton J.L., Meyer T.J. (2009). Making oxygen with ruthenium complexes. Acc. Chem. Res..

[57] Sala X., Romero I., Rodriguez M., Escriche L., Llobet A. (2009). Molecular catalysts that oxidize water to dioxygen. Angew. Chem. Int. Ed..

[58] Schley N.D., Blakemore J.D., Subbaiyan N.K., Incarvito C.D., D’Souza F., Crabtree R.H., Brudvig G.W. (2011). Distinguishing homogeneous from heterogeneous catalysis in electrode-driven water oxidation with molecular iridium complexes. J. Am. Chem. Soc..

[59] Maeda K., Higashi M., Lu D., Abe R., Domen K. (2010). Efficient nonsacrificial water splitting through two-step photoexcitation by visible light using a modified oxynitride as a hydrogen evolution photocatalyst. J. Am. Chem. Soc..

[60] Zou Z., Ye J., Sayama K., Arakawa H. (2001). Direct splitting of water under visible light irradiation with an oxide semiconductor photocatalyst. Nature.

[61] Nocera D.G. (2009). Personalized Energy: The home as a solar power station and solar gas station. ChemSusChem.

[62] Hoganson C.W., T. Babcock G.T. (1997). A Metalloradical mechanism for the generation of oxygen from water in photosynthesis. Science.

